# Sample preparation method for large scale shear testing of soft-clay and granular-column composites

**DOI:** 10.1016/j.mex.2020.100939

**Published:** 2020-05-28

**Authors:** Cihan Cengiz, Erol Guler

**Affiliations:** a University of Birmingham, National Buried Infrastructure Facility, Birmingham, United Kingdom; b Bogazici University, Istanbul, Turkey

**Keywords:** Clay bed, Consolidation, 1-g tests

## Abstract

This article is aimed at giving an overview of the testing equipment and sample preparation techniques for large scale shear testing of granular column enhanced soft clays. A novel testing device, designated Unit Cell Shear Device, is produced to enable clay bed consolidation and subsequent granular column installation within the clay bed. The device allows for studying the shear resistance of unit cells enhanced with slender vertical inclusions such as stone columns, geosynthetic encased stone columns, grouted piles, and conventional piles. The present study illustrates the novel clay slurry consolidation method, sample preparation sequence, testing device features, and the details of the actuators that shear the specimen. The strength parameters of the consolidated clay and the spatial distribution of engineering properties of clay are studied with different testing methods. The techniques prescribed herein present a viable method of clay bed preparation for laboratory testing purposes with the following advantages:

*• Homogeneous clay beds for testing*
*• Reduced drainage path and accelerated consolidation time*
*• Consolidation around a vertical inclusion to allow for column placement*

## Graphical Abstract

**Figure:**
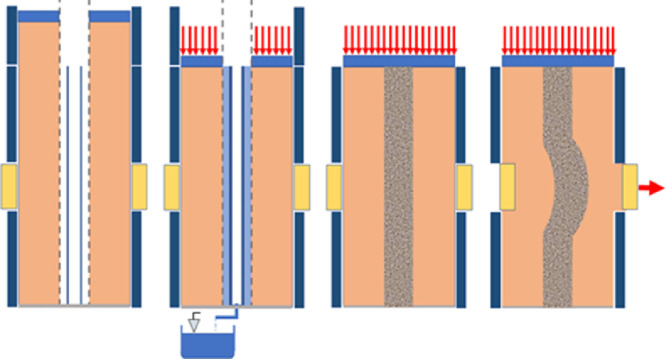

Specifications Table

**Table:** 

Subject Area	Engineering
More specific subject area	*Geotechnical Engineering*
Method name	*Clay sample preparation for large scale laboratory tests*
Name and reference of original method	
Resource availability	

## Introduction

In ground engineering practice, Ordinary Stone Columns (OSCs) and Geosynthetic Encased Columns (GECs) are utilized as a cost effective soil improvement technique where the bearing capacity of the foundation soils are not sufficient to support the vertical loads imposed by the super-structure. Construction of embankments to support infrastructure over soft clay strata is a particularly challenging engineering task where granular column inclusions provide significant advantages. Engineering benefits of granular column inclusions as described by Indraratna et al. [9] include: (i) transmission of foundation loads to a greater depth by a combination of side resistance and end bearing, (ii) reduction in the total and differential settlements, (iii) reduction of liquefaction potential of fine grained soils, and (iv) decreased drainage path for rapid consolidation of soft soil through radial consolidation under foundation loading.

The economic viability and ease of application of granular columns led to an exponential increase in their utilization as foundation elements under challenging design conditions where the nature of the loading on the columns have evolved from static vertical loads to dynamic loads with horizontal components. While the use of the columns have evolved and the loading combinations on the columns have changed, the test methods have not kept up with the industrial demand in being able to accurately quantify the contribution of columns to shear resistance of the host soil under static and dynamic load cases.

Recent applications have further exploited the potential of granular columns where Geosynthetic Encased Columns are used as lateral pressure relief systems where columns withstand lateral loads [16]. Moreover, granular columns are proposed as a foundation system to mitigate seismic loads in rocking foundation applications [11]. Design scenarios as such necessitate a thorough understanding of the granular column enhanced soft soil behavior under lateral loads.

Thanks to the vast amount of experimental work, the behavior of granular columns under static vertical loads are reasonably well understood Mohapatra et al. [13]. Extensive work done around the behavior of column enhanced soils has led to many empirical and analytical calculation methods which are currently utilized in design practice. The behavior of the soil composite formed by column addition under horizontal shear loads on the other hand is not studied in detail, primarily due to the lack of experimental rigs and sample preparation procedures.

Although, the available works in the literature [15,18] have shed some much needed light on the behavior of the soil composite, results of experimental campaigns utilizing samples with the following properties are needed to understand the shear behavior of granular columns embedded in soft soils and extend these findings to well established analytical models for design:

• Correct slenderness ratios: Slenderness ratios that are representative of the field prototypes (height to diameter ratio equal or greater than 10)
• Reinforcement materials: Building the experimental model columns with reinforcement materials that are identical to field counterparts.

The Unit Cell Shear Device (UCSD) is conceived with the intention of serving the above stated purpose by isolating a model unit cell of appropriate size to enable the study of response of the unit cell to shear loading under controlled conditions. The UCSD is designed to enable sample preparation and testing of the soil composite and the device has provisions for instrumentation. In this study the methods of sample preparation for shear testing of granular column enhanced soft ground will be elaborated.

## Conceptualization of Unit Cell Shear Device

**Fig. 1:**
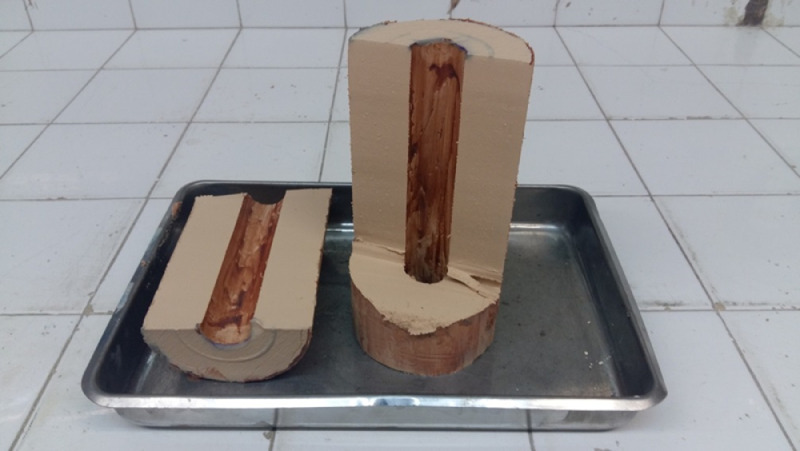
Clay specimen consolidated around a centric vertical void for large scale triaxial testing by [4].

**Fig. 2:**
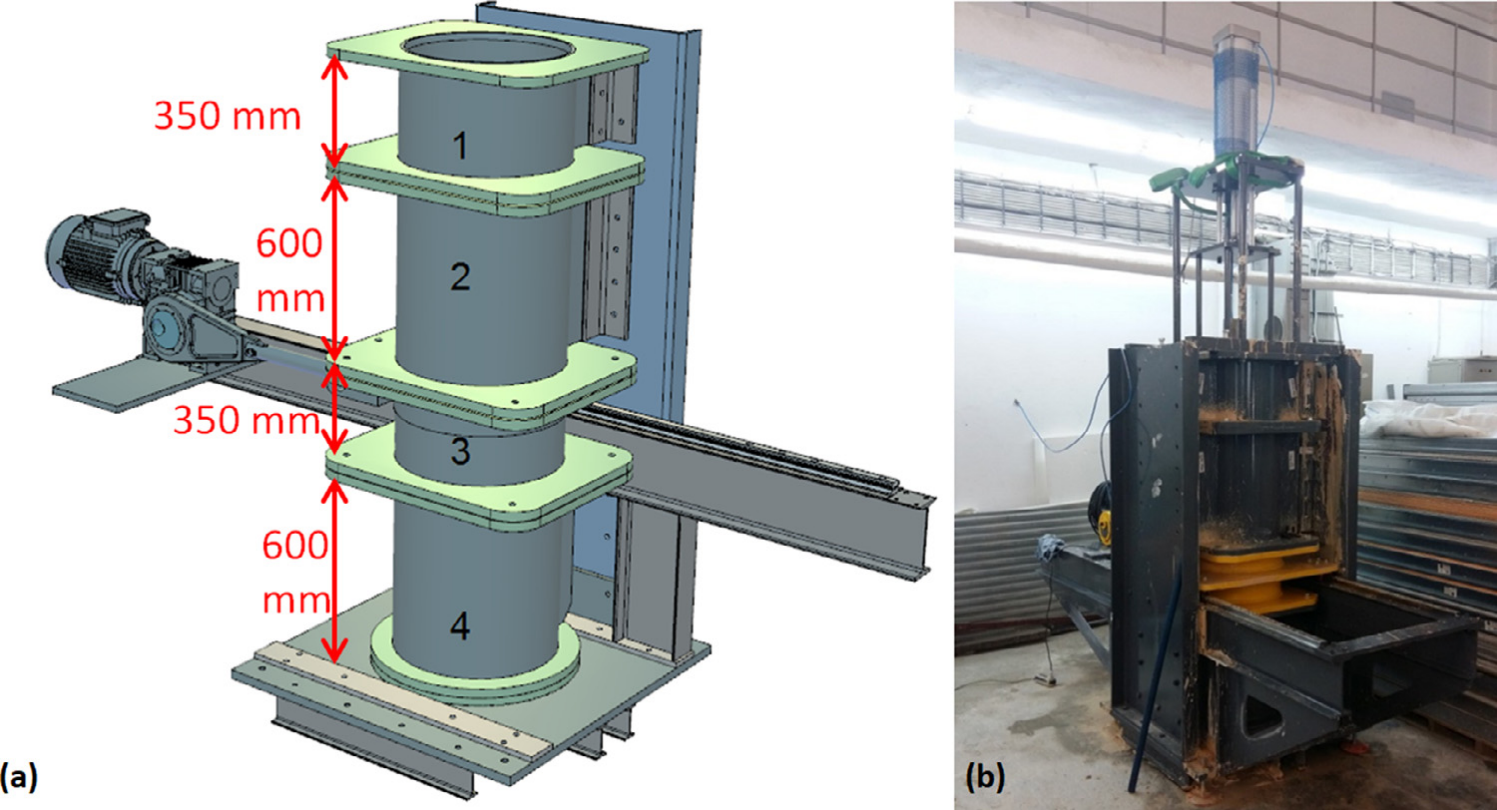
(a) Unit Cell Shear Device CAD sketch illustrating the numbers given to consolidation chambers and their depths in mm and [5] (b) assembly during consolidation of clay slurry.

UCSD is a large scale experimental assembly that allows for consolidation of a clay bed within a cylindrical sample containing volume. Inspired by the earlier laboratory work conducted by the authors [4], the assembly allows for the consolidation of the clay around a vertically oriented shaft which creates a void at the center of the clay bed. A photograph depicting the consolidated clay specimen by Cengiz et al. [4] is illustrated in Fig. 1. The volume created within the clay bed is then used for the placement of a granular column for further testing. Similar to the sample illustrated in Fig. 1 with a diameter and height of 100 and 200 mm, UCSD allows for consolidation of clay beds with a diameter and a height of 460 and 1500 mm. Fig. 2a illustrates the UCSD components on a sketch. The device consolidates the clay around a stainless steel insert with a diameter of 113 mm for granular column placement. Once the sample is prepared, UCSD is capable of shearing the specimen either statically or dynamically. A photograph of the UCSD during shear testing of clay is illustrated in Fig. 2b.

Granular columns can fail in various modes. The columns without encasement sleeves and columns with lower-bound encasement stiffnesses typically fail total rupturing of the column following the geometry of the slip plane. Columns with higher modulus encasements however, do not undergo rupture. Column could also fail in overall bending [6]. UCSD can mimic all failure modes of columns except for overall bending failure. Interested readers are referred to works of Mohapatra et al. [13] and Cengiz et al. [5] for in-depth explanations of possible failure modes for granular columns.

## Unit Cell Shear Device Parts

### Surcharge Assembly

**Fig. 3:**
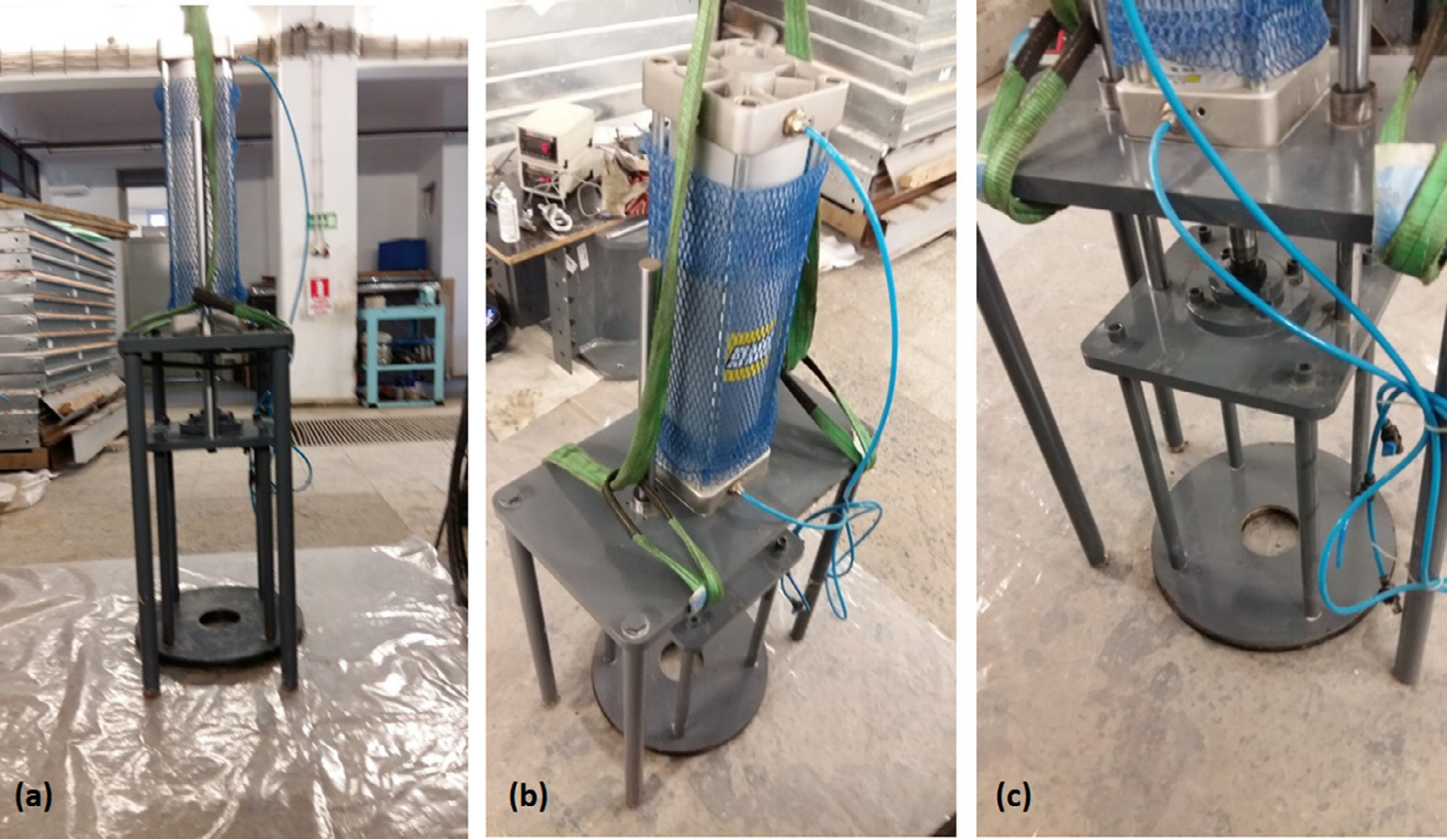
Photographs of surcharge assembly depicting (a) side view, (b) 700 mm stroke 160 mm diameter pneumatic actuator, and (c) doughnut shaped surcharge plate.

The surcharge assembly allows for application of vertical pressures to the clay bed to facilitate consolidation of the clay slurry. The assembly is consisted of a 160 mm diameter pneumatic piston with a 700 mm stroke capacity, a doughnut-shaped surcharge plate, and provisions for reacting the assembly against the experimental rig. Fig. 3a and 3b illustrate side and top views of the assembly. The force output derived from the pneumatic actuator is transferred to the square flange seen in Fig. 3c. Two guide rods extending from the square flange are used to ensure that the plate does not deviate from its vertical axis or twist as the surcharge is applied. As the drainage pipe is situated in the center of the circular surcharge plate, downwards force has to be applied around the center of the surcharge plate. Four steel rods are used to transfer the downwards force to the surcharge plate while allowing a vertical clearance for the drainage pipe. The clay slurry is consolidated around a cylindrical stainless-steel drainage pipe assembly placed in the geometric center of the sample holding volume made up of four 460-mm-inner-diameter consolidation chambers (numbered 1 to 4 in Fig. 2a). The surcharge assembly reacts against the rest of the assembly and it is connected through the threaded indentations machined to the ends of its four legs. The legs are bolted to the assembly to resist the uplifting during surcharge application. The present experimental setup relies on air pressure to apply surcharge loads. With 800 kPa of available air pressure, the surcharge assembly was able to apply 100 kPa of vertical surcharge pressure on the top of the clay bed. Although this value is not typically as high as the vertical stresses in triaxial tests, for the purposes of modelling the loads on soft clay beds at model scale, the pressure applied is adequate.

### Consolidation Chambers

**Fig. 4:**
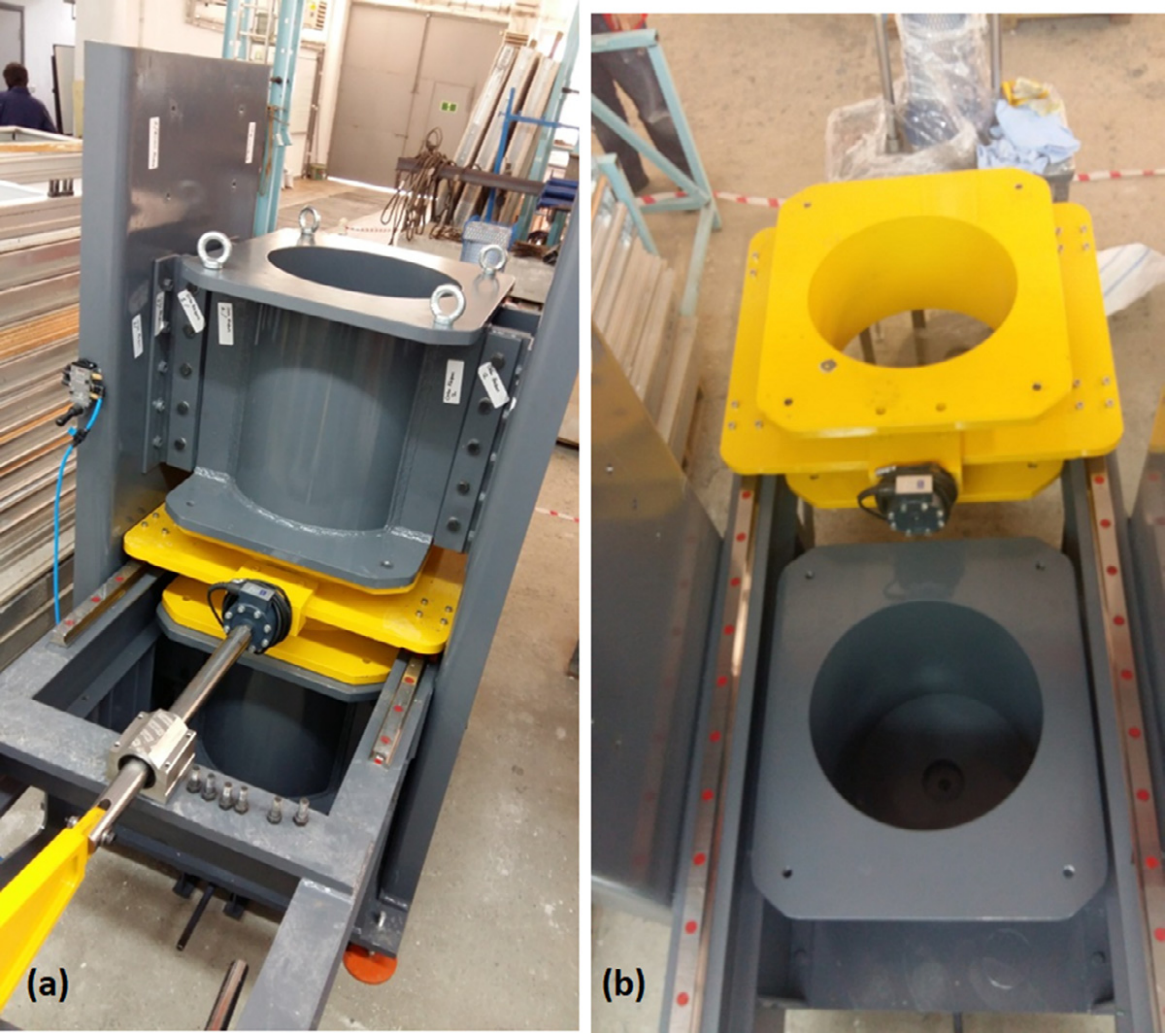
Photographs of the consolidation chambers.

The clay slurry is consolidated inside the sample holding volume created with stacking the multi-layer consolidation chambers above each other. There are a total of four consolidation chambers each with an inner diameter of 460 mm and heights of 350, 600, 300, and 600 mm as depicted in Fig. 2a. The topmost consolidation chamber (number 1 in Fig. 2a) is used to accommodate the consolidation settlement that occurs as the clay bed is subjected to overburden pressures. The clay slurry is filled to the assembly to a height so that the target height of the clay bed remains flush with the top of chamber 2 at the end of consolidation. Chambers 2 and 4 are 600 mm deep with the intent of completely housing the smear effects caused due to the shearing achieved by lateral movement of chamber 3. Chamber 3 is connected to either a static or a dynamic horizontal actuator to shear the consolidated clay bed at mid-height. The chambers are supported by elliptical screw holes on the side walls. This caters for tightly bolting the chambers during consolidation to prevent leakage of the clay slurry. Upon completion of consolidation, the chambers are moved vertically to allow for a gap of 2 mm between the faces of chamber 3 and neighboring chambers. The chambers are also bolted to one another by making use of the clear and threaded holes on the steel skirts that are welded flush with the chamber edges. Chamber 4 sits on a circular inner base that allows for the slight vertical movement of the chamber without causing leakage. Chamber 3 is supported on four load and moment bearing riders that glide freely on stainless steel tracks which has minimal resistance to lateral movements. Once the shear forces are applied to chamber 3, it can displace on the tracks. Fig. 4(a) illustrates chambers 2, 3, 4. Fig. 4(b) illustrates a photograph of the chamber 3 moved along the track and the basal chamber 4.

### Drainage pipe assembly

**Fig. 5:**
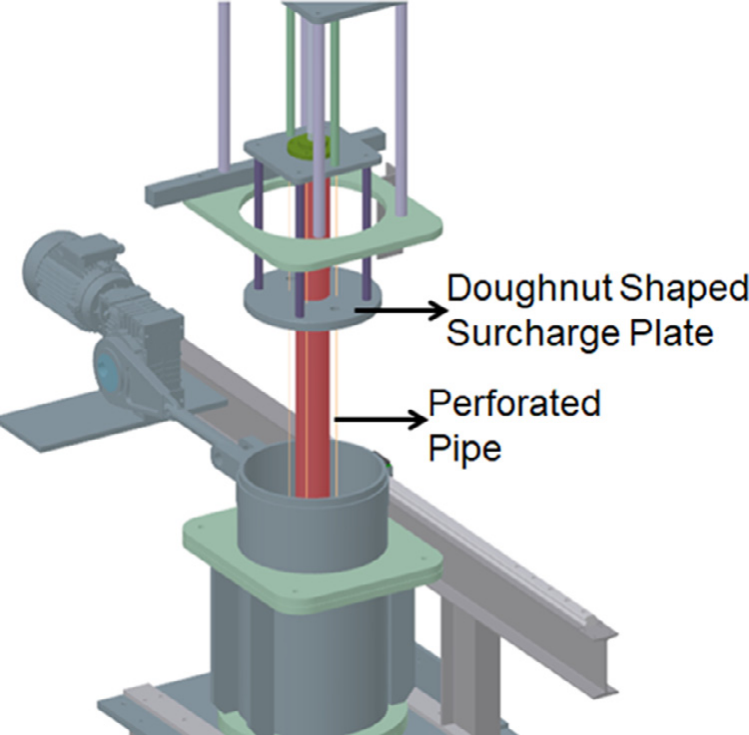
Partial CAD drawing of the parts involved in clay slurry consolidation, doughnut shaped surcharge plate for stress application and perforated pipe used for establishing a cylindrical cavity and drainage of excess water.

The clay slurry is filled around a perforated pipe which serves a dual purpose. The perforated pipe, depicted in Fig. 5, allows for reduced drainage path and consolidation times for the clay slurry. Perforated pipe also allows for a cylindrical cavity at the center of the clay slurry for granular column placement. Once the consolidation is completed the cavity left behind by the drainage pipe assembly is used for placement of granular column inclusions. The surcharge load is applied around the drainage pipe assembly with the doughnut-shaped surcharge plate. A rubber gasket is fitted to the outer periphery and inner opening of the doughnut-shaped surcharge plate to stop any leakage of clay. The drainage pipe assembly is erected on a 50 mm deep machined grove at the base of the assembly. The drainage pipe assembly also has two O-ring bearing grooves in its periphery where it connects to the base of the assembly. The connection is also treated with high vacuum grease to stop any leakage. The vertically oriented void created by the drainage pipe can later be used to install granular columns. Typically, columns without encasement sleeves are installed in lifts of compacted infill material. Encased columns on the other hand require the placement of the encasement in the void geometry prior to infill and subsequent compaction of the infill. A detailed column installation procedure in clay beds is outlined in previous studies of the authors [3,4].

Two concentric stainless steel pipes are used to construct the drainage pipe assembly. The outer pipe is a perforated pipe with an outer diameter of 113 mm. Inside the perforated pipe, a 50-mm-outher diameter pipe is placed. The reason for the placement of the non-perforated pipe is to form a pool of water between the two pipes during consolidation to prevent drying of the clay slurry. The slight extension of the inner pipe ascertains that the clay slurry is inundated under water at all times during consolidation. In the onset of the consolidation, the cavity between two pipes is filled flush with tap water. Upon application of surcharge pressure, the excess interstitial water seeping out of the clay slurry is allowed to drip down from the inner pipe to a collection dish.

## Specimen Preparation

**Fig. 6:**
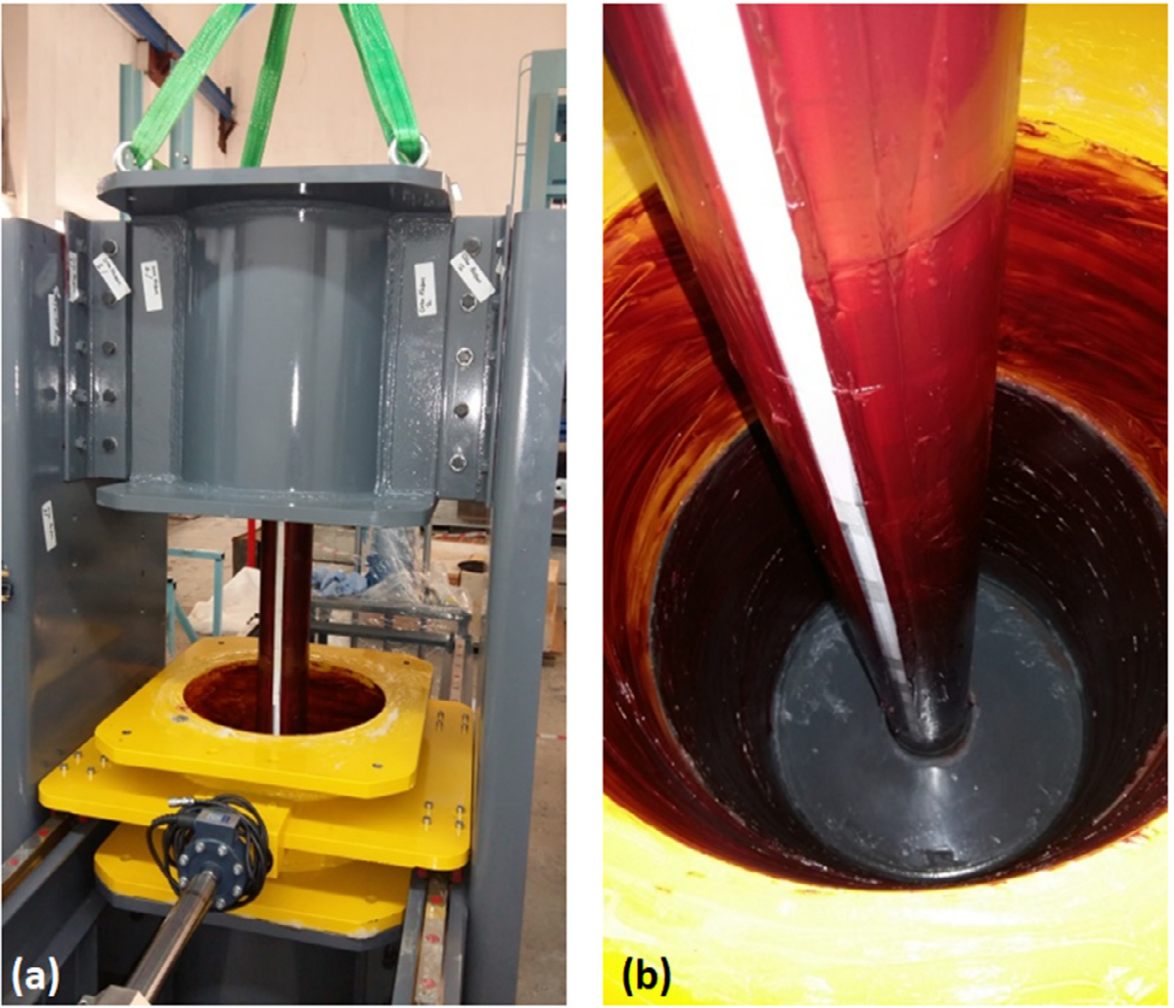
Preparation of the rig for consolidation (a) placement of greased consolidation chamber #2 and greased interior of the assembly.

The inner periphery of the consolidation chambers and the perforated drainage pipe are covered with grease so as to reduce the friction. Exhaustion of vertical surcharge due to side friction is a commonly encountered problem in consolidation dishes with a high depth to diameter ratio. The vertical pressure is countered by side friction and as a result the clay at the lower portions of the consolidation dish experiences a lower value of overburden pressure which leads to variability of clay strength throughout the height of the specimen. Cengiz et al. [4] reported satisfactory results on large scale triaxial testing with grease treated sample container use. Before the perforated pipe illustrated in Fig. 6 is treated with grease, the perforations which are machined in four distinct vertical alignments are covered with filter paper. The filter paper is then covered with a single line of tape to prevent blinding of the material with grease as the material needs to be permeable to pass excess water through the perforations during consolidation. Upon completion of greasing process, the tape is carefully removed. A photograph depicting the grease treated perforated pipe and inner periphery of the testing assembly is illustrated in Fig. 6.

Widely adopted clay bed preparation methods for laboratory testing of stone columns include moulding [1,8], moist tamping [2,17] and slurry consolidation [3,6]. While all of these methods are viable techniques of sample preparation, slurry consolidation helps to ascertain uniformity of strength parameters and uniform spatial distribution of water content throughout the sample [10].

In light of the above, slurry consolidation was chosen as the clay bed preparation method. The slurry preparation commenced by addition of water to a mixing tank and subsequent addition of powder kaolinite clay. The clay slurry is prepared at a water content of 75 % which corresponds to 1.5 times the liquid limit of the kaolinite clay used. Various studies [7,14] in the literature make use of a water content of 1.5 for slurry preparation to avoid formation of lumps in the clay slurry. The tank housing the clay slurry is elevated by an overhead crane and its contents are allowed flow into the consolidation chambers by gravity. This way the consolidation chambers are filled bottom-up to ascertain that no air bubbles are trapped during filling.

Once the infill of the clay slurry was completed, the surcharge assembly was fitted and doughnut shaped surcharge plate was secured on top of the clay slurry surface. The surcharge pressure was applied by incrementally increasing the air flow to the 700-mm-stroke pneumatic actuator by making use of a precision air regulator. An air pressure sensor was placed at the output of the air regulator which was connected to a digital gauge for visual inspection of the applied pressure. The clay beds are then consolidated to overburden pressures of 30, 45, 60, 75, and 90 kPa.

## Shear Testing of the Specimens

**Fig. 7:**
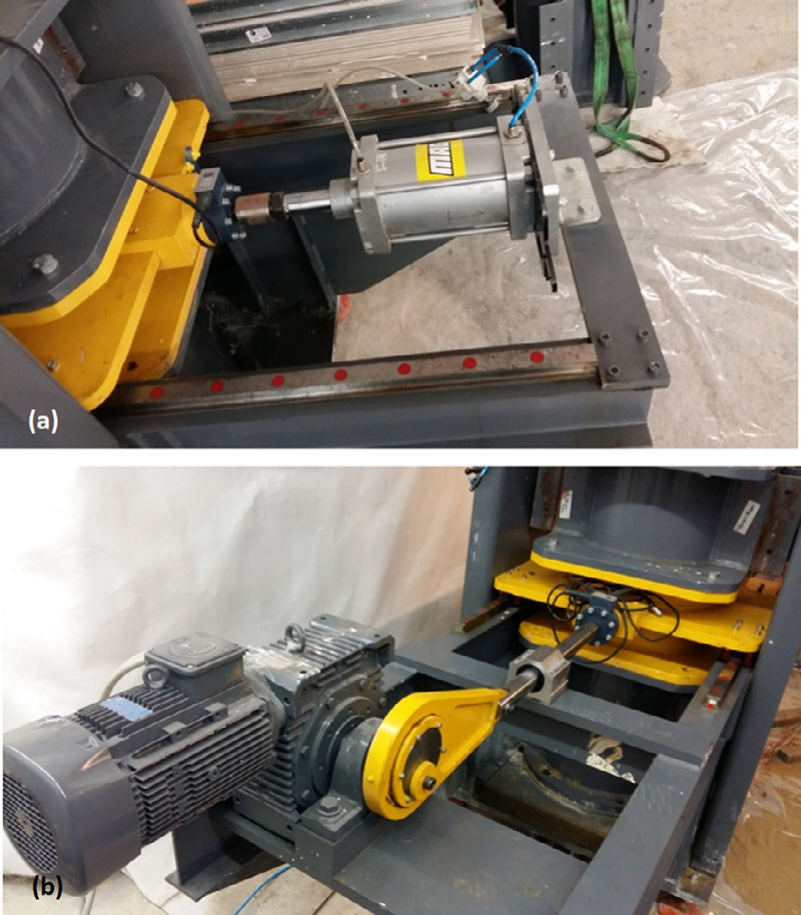
The stress controlled static and displacement controlled dynamic actuators used for shearing the unit cell as depicted by Cengiz et al. [5].

Cengiz et al. [5] have utilized UCSD to undertake shear strength testing of geo-composites formed by granular column addition. The granular column bearing host soil in this study was chosen as sand as the aim of the study was to investigate the effects of GEC and OSC addition on the shear strength parameters of the host soil. UCSD allows for two main types of shearing loads to be applied to the specimens: Stress-controlled static shear and displacement controlled dynamic shear. The static and dynamic shearing units are illustrated in Fig. 6, Fig. 7, respectively. The stress-controlled static shear is achieved by mounting a 160 mm diameter pneumatic actuator with a stroke capacity of 160 mm on the assembly. The drive rod is connected at the mid-stroke of the actuator to allow for 80 mm shearing of the specimen. With a specimen diameter of 460 mm, ± 80 mm shear displacement in either direction corresponds to a shear strain of 17 % which is more than the commonly accepted failure strain in large scale direct shear tests [19]. Dynamic shear loads are applied by making use of an electric motor and a redactor. A Scotch-yoke mechanism is utilized to drive the loading rod axially. Cranks with different off-center pin distances apply cyclic loading displacements of ± 5, 10, 15, 20, 25, 30, and 35 mm at a loading frequency of 1 Hz. Present experimental setup is capable of exciting the soil models at a fixed loading frequency of 1 Hz which is sufficiently close to the resonant frequencies of very soft clay beds [3]. The device also enables application of a wide spectrum of lateral shear displacements which can be altered to fit the requirements of the modelled geotechnical phenomena.

## Results

In order to investigate the viability of the above described apparatus and sample preparation technique for preparation of homogeneous clay samples a series of tests were conducted. Within the scope of the present study, engineering properties of kaolinite clay consolidated to 5 different overburden pressures are investigated. Three types of shear strength tests are conducted to study the properties of the clay beds. These tests are Field Vane tests, Thor-Vane Tests, and unconfined compression tests. Field Vane tests are conducted at various depths of the clay beds by making use of the extension rods of the Field Vane kit which enable the penetration of vane blades into the clay sample. Thor-Vane tests are conducted at the topmost layer of the clay beds. Unconfined compression tests are run by making use of the trimmed samples upon disassembly of the consolidated clay at the end of tests. All of the tests prescribed above are utilized to assess the shear strength properties of the clay beds upon completion of consolidation.

**Fig. 8:**
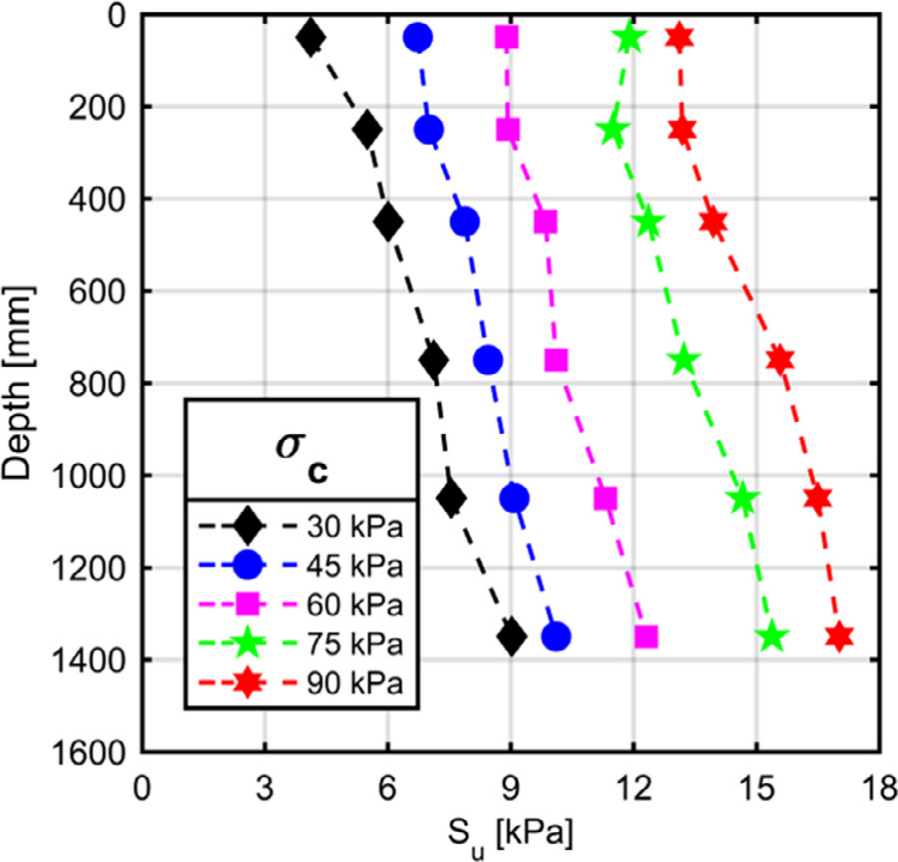
Results of Vane shear tests.

Uniformity of clay beds prepared in a large consolidation tank article has been investigated by Mir et al. [12]. Similar to this study, a series of vane shear tests have been undertaken to determine the variation of strength parameters of the clay bed inside UCSD. A series of Thor-Vane tests are conducted to study the undrained shear strength variation of the clay beds with depth. Fig. 8 illustrates the shear strength measurements taken at discrete locations within the clay beds consolidated to different overburden pressures. As can be seen from the curves illustrated in Fig. 8, the variation of undrained shear strength of the specimens at the topmost measurement location and at the base of the specimens was about 3 kPa. This value sits well with the calculated additional surcharge due to self-weight of the clay body which is about 16 kPa and it is expected to increase the undrained shear strength of the resulting clay by about 3 kPa. It could be said that the samples are prepared with minimal variation of strength properties over the height of the clay beds.

**Fig. 9:**
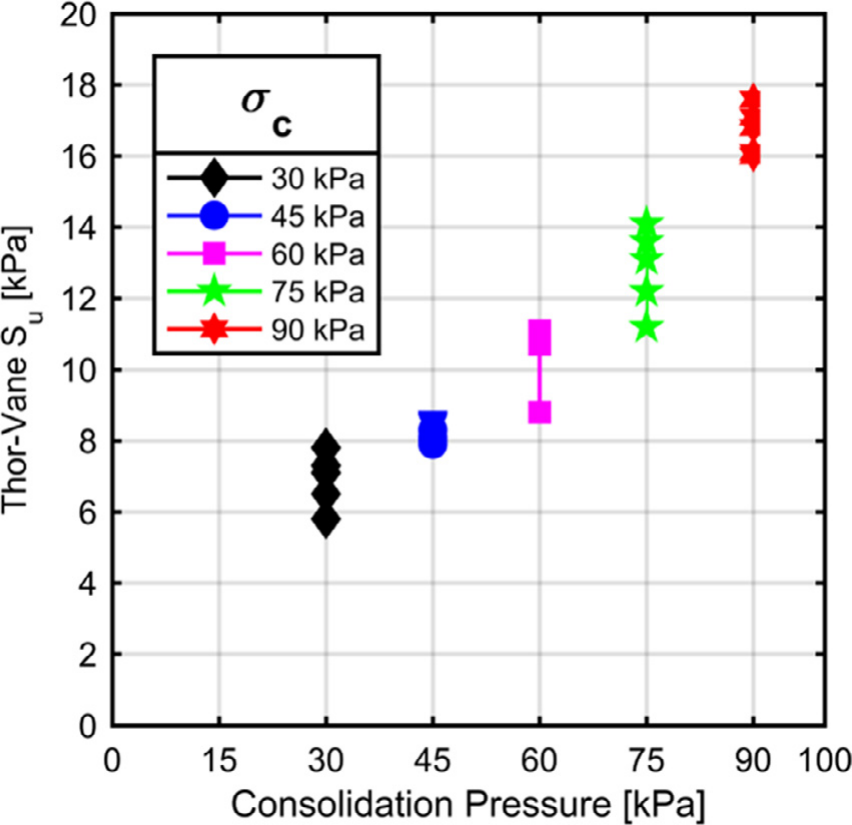
Results of Thor-Vane tests at the topmost level of the clay beds.

**Fig. 10:**
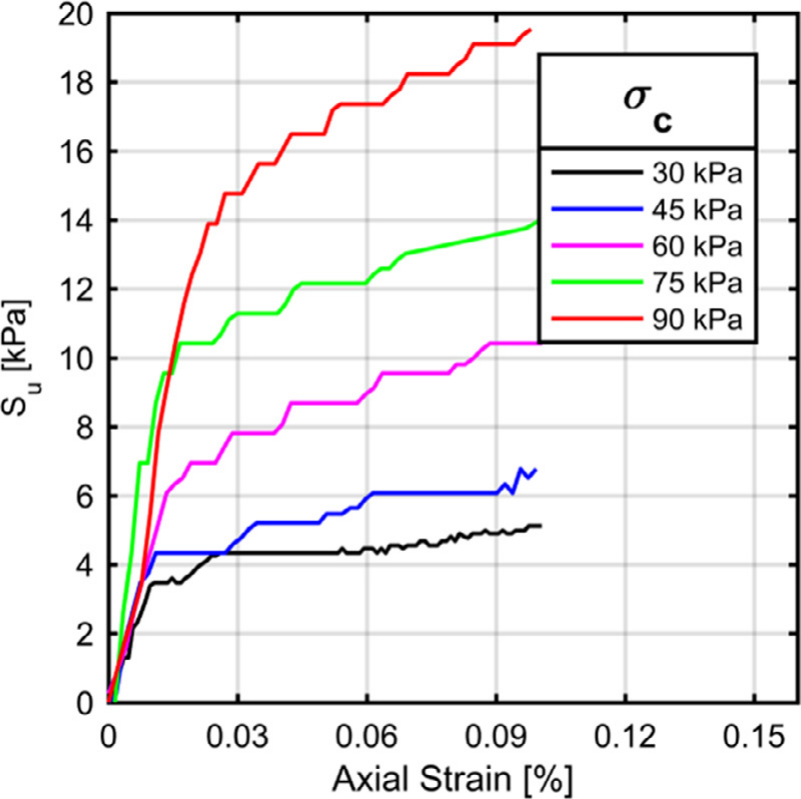
Results of unconfined compression tests.

The Thor-Vane shear tests indicate a linear trend between the applied overburden pressure and undrained shear strength of clay. The Thor-Vane tests are conducted at the topmost plane of the clay beds and the results of these tests are in agreement with the Vane shear tests conducted at a shallow depth as can be seen in Figure 9. Moreover, the unconfined compression tests conducted on trimmed clay samples provide further guidance into the shear strengths of the clays consolidated to different overburden pressures (Figure 10).

Measured undrained shear strength parameters within the clay bed making use of different measurement methods and testing techniques are in close agreement with each other. Widely accepted consolidation pressure to undrained shear strength ratio for kaolinite clays is 0.2. The ratio calculated with different testing techniques are 0.2 for Vane, 0.3 for Thor-Vane and 0.23 for unconfıned compression tests.

Following the 90 kPa consolidation tests, a series of Thor Vane tests were conducted at the interface of second and third consolidation chambers with different testing distances to the drainage pipe assembly. No significant difference in spatial distribution of shear strength was observed in shear strength of the clay which stands to reason that the testing assembly allowed minimal spatial variation of shear strength properties upon completion of consolidation. Thor-Vane testing was chosen to able to accurately pinpoint the location (distance away from the face of the drainage assembly) of testing.

## Conclusions

The UCSD assembly was utilized to prepare clay beds for laboratory testing by making use of a novel consolidation method. The main conclusions drawn from the sample preparation campaign are as follows:

- UCSD test apparatus provides a test bed for large scale physical modelling of slender geotechnical elements withstanding static and dynamic shear loads.
- The device allows for consolidation of clay specimens around a vertically oriented shaft which has provisions to shorten drainage path of the pore water thereby accelerating the consolidation process.
- The spatial variability of consolidated clay specimen's engineering properties such as shear strength are not significantly different apart from the additional surcharge caused by the self-weight of the clay material.
- The undrained shear strength of the kaolinite clay at mid-height of the specimen for overburden stresses of 30, 45, 60, 75, and 90 kPa were 7.1, 8.4, 10.1, 13.2, and 15.6 kPa, respectively.
- Shear strength of the clay determined with different methods are in reasonable agreement where the Thor Vane tests conducted at the topmost layer of the clay beds yielded similar results to that of the (Field) Vane tests.
- Undrained shear strength to consolidation pressure ratio calculated by different test methods were also within reasonable agreement. The ratio calculated with different testing techniques are 0.2 for Vane, 0.3 for Thor-Vane and 0.23 for unconfıned compression tests.
- It was seen that the radial distance of the clay from the drainage pipe did not introduce any spatial variation of shear strength properties.

## Declaration of Competing Interest

None
